# Evolutionary divergence times in the Annonaceae: evidence of a late Miocene origin of *Pseuduvaria *in Sundaland with subsequent diversification in New Guinea

**DOI:** 10.1186/1471-2148-9-153

**Published:** 2009-07-02

**Authors:** Yvonne CF Su, Richard MK Saunders

**Affiliations:** 1 Division of Ecology & Biodiversity, School of Biological Sciences, The University of Hong Kong, Pokfulam Road, Hong Kong, PR China

## Abstract

**Background:**

Phylogenetic analyses of the Annonaceae consistently identify four clades: a basal clade consisting of *Anaxagorea*, and a small 'ambavioid' clade that is sister to two main clades, the 'long branch clade' (LBC) and 'short branch clade' (SBC). Divergence times in the family have previously been estimated using non-parametric rate smoothing (NPRS) and penalized likelihood (PL). Here we use an uncorrelated lognormal (UCLD) relaxed molecular clock in BEAST to estimate diversification times of the main clades within the family with a focus on the Asian genus *Pseuduvaria *within the SBC. Two fossil calibration points are applied, including the first use of the recently discovered Annonaceae fossil *Futabanthus*. The taxonomy and morphology of *Pseuduvaria *have been well documented, although no previous dating or biogeographical studies have been undertaken. Ancestral areas at internal nodes within *Pseuduvaria *are determined using dispersal-vicariance analysis (DIVA) and weighted ancestral area analysis (WAAA).

**Results:**

The divergence times of the main clades within the Annonaceae were found to deviate slightly from previous estimates that used different calibration points and dating methods. In particular, our estimate for the SBC crown (55.2-26.9 Mya) is much younger than previous estimates (62.5-53.1 ± 3.6 Mya and ca. 58.76 Mya). Early diversification of *Pseuduvaria *was estimated to have occurred 15-8 Mya, possibly associated with the 'mid-Miocene climatic optimum.' *Pseuduvaria *is inferred to have originated in Sundaland in the late Miocene, ca. 8 Mya; subsequent migration events were predominantly eastwards towards New Guinea and Australia, although several migratory reversals are also postulated. Speciation of *Pseuduvaria *within New Guinea may have occurred after ca. 6.5 Mya, possibly coinciding with the formation of the Central Range orogeny from ca. 8 Mya.

**Conclusion:**

Our divergence time estimates within the Annonaceae are likely to be more precise as we used a UCLD clock model and calibrated the phylogeny using new fossil evidence. *Pseuduvaria *is shown to have dispersed from Sundaland after the late Miocene. The present-day paleotropical distribution of *Pseuduvaria *may have been achieved by long-distance dispersal, and speciation events might be explained by global climatic oscillations, sea level fluctuations, and tectonic activity.

## Background

The Annonaceae are a large pantropical family of flowering plants, consisting of ca. 135 genera and ca. 2,500 species in predominantly tropical and subtropical lowland forests [1]. The phylogeny of the family has previously been reconstructed based on morphological [2-4] and molecular data [5-7]. Four main clades are consistently recognised in the molecular analyses: two of these clades (consisting of *Anaxagorea *and the small 'ambavioid' clade) form a heterogeneous basal grade, basal to two large clades known as the 'long branch clade' (LBC) and 'short branch clade' (SBC) to reflect differing rates of nucleotide substitutions [7,8].

Evolutionary divergence times based on molecular data have been estimated for the Annonaceae as a whole [5,7] as well as for several individual genera and clades, including *Anaxagorea *[9], 'Andean-centred' genera in the SBC [8], *Guatteria *[10], and an African clade including *Isolona *and *Monodora *[11]. These studies were based on between two and five commonly used chloroplast regions (*matK*, *trnL-F*, *trnT-L*, *rbcL*, and *psbA-trnH *spacer). Comparatively few fossil calibration points were used in these studies due to the scarcity of unequivocal Annonaceae fossils. The most widely applied calibration point is the stem age of the Magnoliaceae, based on the fossil *Archaeanthus *from North America, which either provides a minimum age of 98 Mya in the early Cenomanian [12], or 100 Mya in the late Albian [13]. Other possible calibration points include: 68 Mya for the split between the ambavioids and the combined LBC-SBC clade, derived using *Anonaspermum *seeds from Nigeria [14]; 112 Mya for the split between the Eupomatiaceae and Annonaceae, using the fossil *Endressinia *[15]; and 120 Mya for core Magnoliales, based on Aptian granular monosulcate pollen [16]. In addition, secondary calibration points have been derived from earlier studies of divergence times, including 82 Mya [17] and 90.93 Mya (unpublished estimate by M.D. Pirie) for the stem of the Annonaceae. Richardson et al. [7] have accordingly estimated the Annonaceae stem age at 90.6 ± 1.3 Mya, based on three calibration points [7]. The age estimates for the family have enabled significant palaeobiogeographical insight, although the most important work has focused on dispersal patterns in Africa and South America (western Gondwana) since the genera studied were largely neotropical.

The program r8s [18] has been widely adopted in studies of divergence times in the Annonaceae [7,9,10]. This program incorporates both non-parametric rate smoothing (NPRS) methods and penalized likelihood (PL) methods. NPRS methods estimate ages through a smoothing criterion [19], whereas PL is a semiparametric approach [20] that combines parametric methods with the robustness of non-parametric methods. Other dating methods utilized in studies of the Annonaceae [21] include PAML [22] and MULTIDIVTIME [23], based on Bayesian dating methods. BEAST (Bayesian Evolutionary Analysis Sampling Trees) [24] is the only software that simultaneously co-estimates phylogeny, node ages and substitution rates. BEAST has recently been used to estimate the origins of the East African lineages within the Annonaceae [11].

The present study focuses on the palaeotropical genus *Pseuduvaria*, which is nested within the SBC. The genus consists of 56 species of trees and treelets (inclusive of *Craibella phuyensis*, which has recently been shown to be congeneric with *Pseuduvaria *[25,26], and three newly described species [26]). A comprehensive monograph of the genus has been completed [27] and the phylogeny of *Pseuduvaria *has been inferred using five chloroplast regions [25]. The genus was previously estimated to have evolved at least ca. 16 Mya ago based upon a published chronogram [7], although this may not represent the true age of the genus since the study included only five *Pseuduvaria *species (inclusive of *C. phuyensis*).

**Figure 1:**
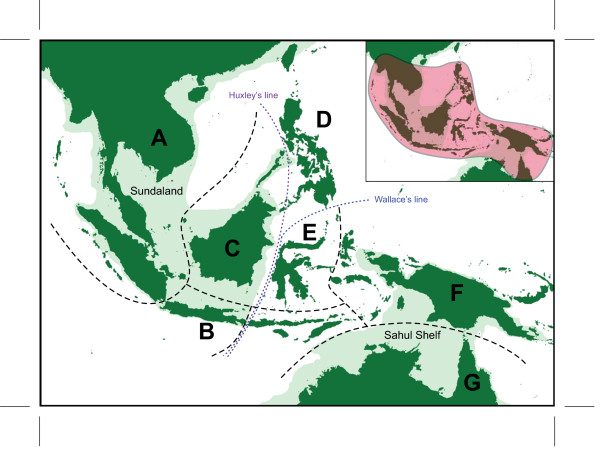
**Main biogeographical areas used in analyses, and (inset) distribution of *Pseuduvaria *in SE Asia**. Eight areas are outlined and labelled: A, China, Indochina, Burma, Thailand, Peninsular Malaysia and Sumatra; B, Java and Bali; C, Borneo and Palawan; D, Philippines (excluding Palawan); E, Sulawesi; F, New Guinea; and G, Australia. The shaded light green area represents shallow continental shelves, as modified from [28].

The Malesian phytogeographical region is separated into two main subregions by Huxley's line and Wallace's line (Figure 1: [28]): western Malesia, which includes Peninsular Malaysia, Sumatra, Java, Borneo and Palawan; and eastern Malesia, which includes Sulawesi, the Lesser Sunda Islands, the Moluccas, and New Guinea. Borneo and New Guinea represent the two most important centres of plant species richness and endemism within Malesia [29]. *Pseuduvaria *is widely distributed from Indochina to New Guinea and NE Australia (Figure 1, inset), with the main centre of diversity in New Guinea (with 20 species) and a secondary centre in Peninsular Malaysia (with 16 species, including three recently described species) [26]. In an earlier molecular phylogenetic study of *Pseuduvaria*, five clades were recognized, with the three basal clades occurring in the western Malesia [25]. A continuous land mass connecting Indochina, Thailand, Peninsular Malaysia, Borneo, Sumatra, and Java (collectively known as Sundaland) was formed during the Early Mesozoic [30], separated from the Australian-New Guinea plate by a major ocean barrier. The collision between the Asian and New Guinea land masses during the Cenozoic may have promoted the dispersal of *Pseuduvaria *species from Sundaland to New Guinea. The present paper aims to use molecular dating techniques to test whether this biogeographical hypothesis is supported by evidence of past geological events.

We use Bayesian molecular dating techniques in BEAST to estimate the divergence times of major Annonaceae clades (including the date of origin of *Pseuduvaria *as well as clades within *Pseuduvaria*). Five chloroplast DNA regions, *psbA-trnH *spacer, *trnL-F*, *matK*, *rbcL *and *atpB-rbcL *spacer, were used in all analyses. The divergence times of major Annonaceae clades were estimated using a dataset ('matrix A') with a broad taxonomic sampling across the basal grade, LBC and SBC of the Annonaceae, together with representatives of related families in the Magnoliales. Two fossil calibration points were used to date the divergence times within the Annonaceae: the fossil *Archaeanthus *[12], providing a minimum age of 98 Mya for the stem of the Magnoliaceae (J.A. Doyle, pers. comm.); and the Late Cretaceous fossil *Futabanthus*, providing a minimum age of 89 Mya for the split between *Anaxagorea *and the combined ambavioid-LBC-SBC clade [31]. The ages of clades within *Pseuduvaria *were subsequently estimated using a smaller dataset ('matrix B') that included all *Pseuduvaria *species available (54 species) and selected members of the SBC. The age estimates inferred from BEAST analyses using matrix A were used as prior information in the subsequent analyses using matrix B. An additional objective was to identify the most likely biogeographical origin of *Pseuduvaria*, and to infer subsequent dispersal patterns using dispersal-vicariance analysis (DIVA) [32] and weighted ancestral area analysis (WAAA) [33].

## Methods

### Taxon sampling and sequence data

We used two separate datasets: matrix A (for higher-level analyses) and matrix B (for species-level analyses within *Pseuduvaria*). Both sets of analyses were based on chloroplast DNA sequences from five regions (*psbA-trnH *spacer, *trnL-F*, *matK*, *rbcL *and *atpB-rbcL *spacer). The materials and methods for DNA extraction, PCR amplification and sequencing of *Pseuduvaria *are detailed in earlier publications [25,26]. The sequences for the outgroups were downloaded from GenBank. The accession numbers for all sequences are presented in Additional File 1.

**Figure 2:**
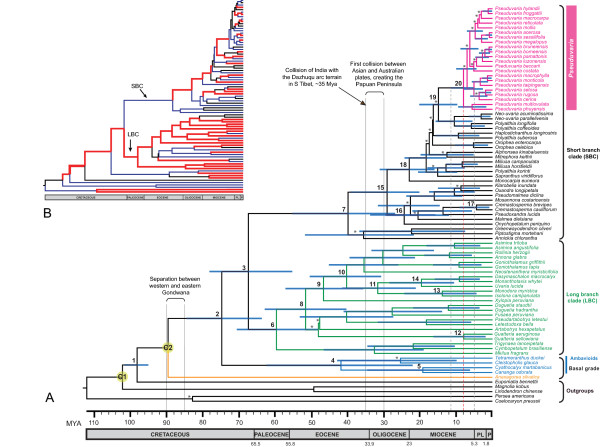
**Chronograms of Annonaceae: maximum clade credibility trees from the BEAST analysis of matrix A**. (A). Posterior estimates of divergence times were inferred using partitioned analyses based on five chloroplast regions, a UCLD model, and two fossils as minimum age constraints: an *Archaeanthus *fossil date of 98 Mya (calibration C1), and a *Futabanthus *fossil date of 89 Mya (calibration C2). Nodes are posterior mean ages (Mya), with blue node bars representing the 95% HPD intervals (see Table 2 for details). Bayesian PP < 0.95 are indicated by asterisks above branches. Geological time-scale abbreviations: PL, Pliocene; P, Pleistocene. The red dashed line represents the estimated tMRCA of *Pseuduvaria *crown, with a 95% HPD indicated by the grey vertical lines. (B). Rate of evolution across all branches. Broad red lines indicate lineages where the posterior rate is greater than the mean rate; narrow blue lines indicate lineages where the posterior rate is lower than the mean rate.

Matrix A comprised a total of 83 species. The ingroup included 22 of the total 56 *Pseuduvaria *species, representing all five main clades identified in our previous phylogenetic study [25], and 56 species from 46 different Annonaceae genera (inclusive of the basal grade, LBC and SBC). The outgroups included were *Coelocaryon preussii *(Myristicaceae), *Eupomatia bennettii *(Eupomatiaceae), *Liriodendron chinense *(Magnoliaceae), *Magnolia kobus *(Magnoliaceae), and *Persea americana *(Lauraceae). Matrix B consisted of 69 taxa, with 54 *Pseuduvaria *species. The selection of outgroups was based on the phylogeny generated by BEAST using matrix A (Figure 2A). The SBC genera selected as outgroups were *Alphonsea*, *Haplostichanthus*, *Miliusa*, *Monocarpia*, *Neo-uvaria*, *Orophea*, *Polyalthia*, and *Sapranthus*, representing 15 species.

For matrices A and B, sequences were edited and assembled in SeqManPro using DNAStar Lasergene 8 (DNAStar), and aligned manually using BioEdit ver. 7.09 [34] and Se-Al ver. 2.0a11 [35]. Ambiguously aligned positions were excluded from the analyses. The combined five-region data sets in matrices A and B are composed of 4860 and 4361 characters, respectively (see Table 1 for data on individual gene regions).

**Table 1:** Length and best-fitting model for data partition analyses based on two different datasets (matrices A and B).

	Aligned length (bp)		Sequence evolutionary model	
	
	Matrix A	Matrix B	Matrix A	Matrix B
*psbA-trnH *spacer	572	441	GTR+G	GTR+G
*trnL-F*	1064	937	GTR+G	GTR+I
*matK*	823	811	GTR+G	GTR+G
*rbcL*	1344	1344	GTR+I+G	HKY+I+G
*atpB-rbcL *spacer	1057	828	GTR+G	GTR+I
Combined data	4860	4361	GTR+I+G	GTR+I+G

GTR, general time-reversible model; HKY, the Hasegawa, Kishino, and Yano model; I, proportion of invariant sites; G, gamma distribution.

### Divergence time estimation

MrModelTest ver. 2.3 [36] was used to determine the appropriate DNA substitution model and gamma rate heterogeneity using the Akaike Information Criterion (AIC). For both matrices, MrModelTest was performed for each gene region and combined regions (Table 1). For the combined five chloroplast DNA regions in matrices A and B, the GTR+I+G was determined as the best-fitting statistical model.

The tree topology, node ages and substitution rates were simultaneously estimated using a Bayesian MCMC (Markov chain Monte Carlo) approach as implemented in BEAST ver. 1.4.8 [24,37]. For matrix A analyses, exponential priors were selected for the two calibration points (see discussion below): the *Archaeanthus *calibration used 98 Mya (labelled C1 in Figure 2A) as zero offset, with an exponential mean of 1; and the *Futabanthus *calibration used 89 Mya (labelled C2 in Figure 2A) as zero offset, with an exponential mean of 1. A Yule speciation tree prior was furthermore specified; this prior has been recommended for species-level phylogenies, and assumes a constant rate of speciation per lineage [24]. An uncorrelated lognormal distributed relaxed clock (UCLD) model was employed, which allows evolutionary rates to vary along branches within lognormal distributions [38]. The five chloroplast regions were partitioned (see Table 1 for the different models of substitution), allowing the incorporation of different models of substitution for each gene independently; the xml input file based on matrix A was edited manually for gene region partition following the tutorial by T.L.P. Couvreur [11,39].

Two independent MCMC runs were performed, each run of 50 million generations, with sampling every 5000 generations. The two separate runs were then combined (following the removal of 10% burn-in) using LogCombiner ver. 1.4.8 [24,37]. Adequate sampling and convergence of the chain to stationary distribution were confirmed by inspection of MCMC samples using Tracer ver. 1.4 [40]. The effective sample size (ESS) values of all parameters were greater than 200, which was considered a sufficient level of sampling. The sampled posterior trees were summarized using TreeAnnotator ver. 1.4.8 [24,37] to generate a maximum clade credibility tree (maximum posterior probabilities) and calculate the mean ages, 95% highest posterior density (HPD) intervals, posterior probabilities (PP) and substitution rates for each node. The BEAST topology (Figure 2A) was visualized with FigTree ver. 1.2 [41]. The MCMC analysis based on matrix A was also performed without sequence data; the prior distribution can therefore be compared with the posterior distribution in order to examine the influence of the data and prior, showing that the results are not influenced by the chosen priors alone [38].

**Figure 3:**
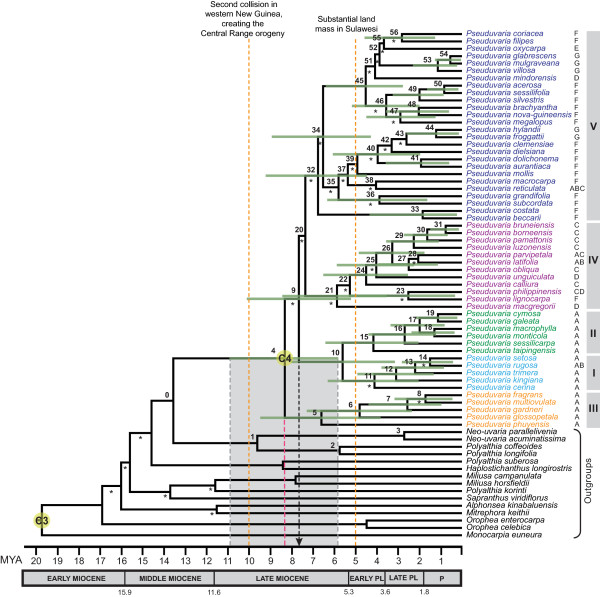
**Chronogram of *Pseuduvaria*: maximum clade credibility tree from the BEAST analysis of matrix B**. Posterior estimates of divergence times were inferred using partitioned analyses based on five combined chloroplast regions, a UCLD model, and two secondary calibration points (labelled C3 and C4). Nodes are posterior mean ages (Mya), with green node bars representing the 95% HPD intervals (see Table 3 for details). Bayesian PP < 0.95 are indicated by asterisks below branches. Letters to the right of taxon names represent current geographical distributions (defined in Figure 1). Geological time scale abbreviations: PL, Pliocene; P, Pleistocene. The red dashed line represents the estimated tMRCA of *Pseuduvaria *crown, with a 95% HPD indicated by the grey bar; black dashed line represents the earliest possible migration event for *Pseuduvaria *out of Sundaland.

The time to most recent common ancestor (tMRCA) of *Monocarpia *and *Pseuduvaria *inferred from the MCMC runs of matrix A were used as prior information in the subsequent BEAST analyses using matrix B in order to estimate the divergence times within *Pseuduvaria *lineages. A normal probability distribution was employed as priors for matrix B analyses, since the 95% HPD intervals from previous estimation (based on matrix A) can also be accommodated as uncertainty for this analysis. The normal probability distribution is thought to reflect uncertainty in secondary calibration points [11,42,43]. Normal distribution priors were therefore applied to calibration points at two nodes: the MRCA of *Monocarpia*, with a mean age of 23 Mya and a standard deviation of 3.78 Mya (labelled C3 in Figure 3); and the MRCA of *Pseuduvaria*, with a mean age of 8 Mya and a standard deviation of 1.64 Mya (labelled C4 in Figure 3). The xml input file based on matrix B was edited manually for partitioned chloroplast sequence data. A UCLD model with Yule speciation tree prior was employed. Two independent MCMC runs of 30 million generations were performed, with sampling every 3000 generations. The two separate runs were then combined (following removal of 10% burn-in) using LogCombiner ver. 1.4.8 [24,37]. The runs were checked for convergence using Tracer ver. 1.4 [40], and the ESS values of all parameters shown to be greater than 500, which was considered sufficient. The BEAST topology (Figure 3) was summarized and visualized as described above.

### Fossil calibrations

The most commonly used calibration point in previous studies of the Annonaceae has been the fossil *Archaeanthus *[7,8] which provides an age of 98 Mya for the stem of the Magnoliaceae. Other calibration points previously employed for the Annonaceae are 120 Mya for the node of the core Magnoliales using the pollen fossil *Lethomasites *[9], 112 Mya for the split between the Eupomatiaceae and Annonaceae using the *Endressinia *fossil [15], and 68 Mya for the common ancestor of the ambavioids and combined LBC-SBC clade using the Maastrichtian seed genus *Anonaspermum *from Nigeria and Sierra Leone [14].

*Lethomasites *was not used in the present study since its placement within the Magnoliales is doubtful [5]. The inclusion of *Lethomasites *would also require broader taxonomic sampling of other families within the Magnoliales (including Degeneriaceae and Himantandraceae), and the large resultant dataset would require considerably longer computational time. The *Endressinia *fossil was not reliable as its relationship with the Eupomatiaceae is doubtful [11]. The *Anonaspermum *seeds were similarly avoided as calibration points in the present study as the position of this fossil within the Annonaceae is uncertain [44].

The inclusion of a greater number of fossil calibration points can reduce bias and result in more accurate age estimates, assuming that the chosen points are mutually consistent [45]. The fossil record of the Annonaceae is very limited, however, and only two fossils, *Archaeanthus *and *Futabanthus*, were regarded as sufficiently reliable. The phylogenetic position of *Archaeanthus *[12] appears to be reliable as it shares several characters with the Magnoliaceae, including distinctive stipules, elongated receptacle and fruits with numerous follicles [5,11,12]. The phylogenetic position of the recently discovered Late Cretaceous fossil flower, *Futabanthus asamigawaensis *[31], is similarly reliable as it shares many morphological similarities with the Annonaceae, including hermaphroditic, hypogynous flowers with six tepals in two whorls of three, an androecium consisting of numerous stamens with flattened connectives that extend above the thecae, and a gynoecium of numerous free carpels. Takahashi et al. [[31]: 914] postulated that *Futabanthus *has "a position within crown-group Annonaceae, perhaps as sister or near the base of all extant taxa except *Anaxagorea*." Inner staminodes are absent in *Futabanthus *but are present in the basal genus *Anaxgaorea*; the lack of inner staminodes is therefore possibly synapomorphic for the rest of the Annonaceae (plesiomorphic in *Anaxagorea*). We set *Futabanthus *as a minimum age constraint of 89 Mya for the split between *Anaxagorea *and the combined ambavioid-LBC-SBC clade (i.e., the crown age of the Annonaceae, labelled as C2 in Figure 2A).

Previous dating studies furthermore utilized secondary calibration points that were extracted from earlier estimates, viz.: the age of 82 Mya for the stem of the Annonaceae based on Wikström et al. [17] using the NPRS method [7,10]; and the age of 90.93 Mya for the stem of the Annonaceae based on unpublished estimations by M.D. Pirie. Secondary calibration points generated by these previous Annonaceae studies were avoided as a different molecular dating method and calibration points were employed in this study.

### Historical biogeography

Ancestral biogeographical areas occupied by *Pseuduvaria *were inferred using two methods: dispersal-vicariance analysis (DIVA) [32] and weighted ancestral area analysis (WAAA) [33]. Eight main biogeographical areas represented in the current distributions of *Pseuduvaria *species were used for the ancestral area reconstructions (Figure 1): (A) southern China, Indochina, Thailand, Burma, Peninsular Malaysia, and Sumatra; (B) Java and Bali; (C) Borneo and Palawan (identified as a single biogeographical unit following [46]); (D) Philippines (excluding Palawan); (E) Sulawesi; (F) New Guinea and the Moluccas; (G) Australia; and also the outgroup distribution (H) India and Sri Lanka (not shown in Figure 1). Species distribution data were derived from our monograph of *Pseuduvaria *[27].

DIVA was implemented using ver. 1.2 of the program [32], in which ancestral distributions are inferred by minimizing the number of dispersal and extinction events. DIVA assumes vicariance is the default mode of speciation, thus it does not assign a cost for vicariance but counts steps for dispersal and extinction events. The tree topology resulting from the BEAST analyses based on matrix B was used as DIVA requires a fully resolved tree. The sister clade with four species (*Neo-uvaria acuminatissima*, *Neo-uvaria parallelivenia*, *Polyalthia coffeoides *and *Polyalthia longifolia*) was also included in the biogeographical analysis. The geographical distributions estimated for internal nodes were optimized by constraining the maximum number of unit areas to two, hence restricting the number of unit areas in ancestral distributions.

WAAA estimates ancestral areas using reversible parsimony, and weights areas in plesiomorphic branches more than apomorphic branches [33]. Probability indices (PI) were calculated for each area at each node by counting the number of weighted gain steps (GSW) and weighted loss steps (LSW) on the cladogram resulting from the BEAST analyses (also based on matrix B). The PI is the ratio of LGW and LSW; biogeographical areas with a value of less than 0.2 are not considered as part of the ancestral area.

## Results

### Phylogeny and age estimations

The BEAST analyses of matrix A resulted in a robust phylogeny of the Annonaceae (Figure 2A), which is largely consistent with the topologies of previous phylogenetic analyses [7] and unpublished phylogenies using MrBayes (based on two and seven genes: L.W. Chatrou, pers. comm.). The mean ages of major nodes, with 95% HPD intervals and Bayesian PP values, are indicated in the chronogram (Figure 2A) and Table 2. The mean rate of evolution is 0.0006 substitutions per site per million years (95% HPD: 5.23E-4-7.10E-4). The 'birth rate' (i.e., speciation rate) indicated by the Yule prior is 0.041 (95% HPD: 0.031–0.052). The coefficient of variation is 0.69 (95% HPD: 0.56–0.84), indicating that substitution rate heterogeneity across the tree and that a relaxed clock model is most appropriate [47].

**Table 2:** Prior and posterior age distributions of major nodes of Annonaceae based on matrix A using BEAST analyses.

Node	Posterior		Prior		Previous estimates	Bayesian PP
			
	Mean (Mya)	95% HPD (Mya)	Mean (Mya)	95% HPD (Mya)		
C1: Magnoliaceae stem age	102.10	106.20-98.0	98.89	100.09-98.00	-	1.00
1: Annonaceae stem age	98.01	101.46-94.91	-	-	90.6 ± 1.3 by NPRS [7], 82 by PL [9]	1.00
C2: Annonaceae crown age	89.46	90.41-89.0	90.05	92.20-89.00	-	1.00
2: split between *Cananga- Tetrameranthus *and both LBC-SBC clade	74.66	84.4-63.62	82.58	91.88-69.53	-	1.00
3: split between LBC and SBC	67.33	78.08-55.22	-	-	66.7-56.6 ± 2.3 by NPRS [7]	1.00
4: *Cananga*-*Tetrameranthus*	41.80	62.35-22.25	39.09	71.91-5.75	51.3-43.8 ± 5.1 by NPRS[7]	1.00
5: *Cananga-Cyathocalyx*	19.45	34.52-6.42	-	-	-	1.00
6: LBC crown node	59.59	70.5-48.07	61.95	85.26-38.61	60.2-51.1 ± 2.3 by NPRS [7]	1.00
7: SBC crown node	39.84	55.17-26.86	70.82	88.09-52.69	62.5-53.1 ± 3.6 by NPRS[7], 58.76 by PL [8]	1.00
8: *Guatteria-Asimina*	51.54	62.81-40.32	-	-	-	1.00
9: *Xylopia-Asimina*	46.62	57.1-35.68	-	-	-	0.97
10: *Isolona-Asimina*	40.23	50.41-30.71	-	-	-	1.00
11: *Isolona-Dasymaschalon*	31.65	41.45-21.18	-	-	-	1.00
12: *Guatteria*	7.94	16.3-1.91	-	-	11.4 ± 1.4 by PL[10]	1.00
13: *Isolona-Monodora*	13.85	24.32-4.43	-	-	Mean = ca. 14 by BEAST [11]	1.00
14: *Uvaria-Dasymaschalon*	19.61	28.42-10.59	-	-	14.8-12.4 ± 2.4 by NPRS[7]	1.00
15: *Onychopetalum-Pseuduvaria*	29.08	39.19-20.18	-	-	-	1.00
16: *Onychopetalum-Karobelia*	24.36	37.75-15.77	-	-	37.65 by NPRS[8], 24.76 by PL[8], 31.81 by Multidivtime [8]	1.00
17: *Crematosperma*	4.44	9.09-0.90	-	-	22.33 by NPRS, 7.17 by PL[8], 16.49 by Multidivtime [8]	1.00
18: *Monocarpia-Pseuduvaria*	22.97	31.14-16.01	-	-	-	1.00
19: *Neo-uvaria-Pseuduvaria*	14.9	20.2-9.7	-	-	-	< 0.95
20: *Pseuduvaria*	8.01	11.5-5.0	52.11	74.09-29.04	ca. 16 by NPRS[7]	1.00

Node numbers refer to Figure 2A. Mean ages and 95% HPD intervals of divergence times are in millions of years before the present. Age estimates were compared with earlier dating studies for the Annonaceae. Bayesian PP are also indicated. C1 and C2 represent the calibration nodes (see texts for details).

The BEAST chronogram (Figure 2A) is composed of four major clades: *Anaxagorea *(sister to all other members of the Annonaceae); a clade consisting of *Cananga*, *Cleistopholis*, *Cyathocalyx *and *Tetrameranthus*; the long branch clade (LBC); and the short branch clade (SBC). Each of these clades received strong statistical support (Table 2: PP = 1.00). BEAST analyses based on matrix A gave an estimated tMRCA for *Pseuduvaria *of 8.0 Mya (95% HPD: 11.5-5.0 Mya, PP = 1.00; Figure 2A: node 20). The MCMC result of the posterior distribution for the tMRCA of *Pseuduvaria *(Table 2: node 20) is shifted ca. 44 Mya later, indicating that the sequence data strongly influences date estimation.

The BEAST analyses of matrix B generated a well-resolved phylogeny of *Pseuduvaria*. The mean ages, 95% HPD intervals and Bayesian PP values of all nodes within *Pseuduvaria*, are indicated in the chronogram (Figure 3) and Table 3. The mean rate of evolution is 0.0005 substitutions per site per million years (95% HPD: 3.57E-4-7.024E-4). The birth rate indicated by the Yule prior is 0.188 (95% HPD: 0.122–0.270). The coefficient of variation is 0.543 (95% HPD: 0.365–0.723), indicating that substitution rate heterogeneity across the tree and that a relaxed clock model is appropriate [47]. The phylogeny of *Pseuduvaria *is segregated into five clades (Figure 3: clades I-V, numbering follows [25]). The BEAST topology of *Pseuduvaria *is largely consistent with those resulting from earlier ML and Bayesian analyses [[25]: Figure 3].

**Table 3:** Posterior estimates of divergence time estimates (Mya) in *Pseuduvaria *based on matrix B using BEAST analyses, with results of ancestral area reconstructions using dispersal-vicariance analysis (DIVA) and weighted ancestral area analysis (WAAA).

Node	Age estimates		DIVA	WAAA								Bayesian
				
	Mean (Mya)	95% HPD (Mya)		A	B	C	D	E	F	G	H	PP
0	13.6	18.49-8.85	AH	0.40	0.08	0.40	0.17	0.03	0.43	0.06	0.33	1.00
1	9.64	14.10-5.29	CH	-	-	1	-	-	-	-	1	1.00
2	5.76	9.37-2.41	H	-	-	-	-	-	-	-	∞	1.00
3	2.72	5.34-0.59	C	-	-	∞	-	-	-	-	-	1.00
4	8.34	10.95-5.82	A	0.93	0.09	0.25	0.29	0.04	0.52	0.07	-	1.00
5	6.62	9.50-3.83	A	∞	-	-	-	-	-	-	-	0.99
6	4.82	7.31-2.36	A	∞	-	-	-	-	-	-	-	1.00
7	2.57	4.40-0.97	A	∞	-	-	-	-	-	-	-	1.00
8	1.72	3.18-0.44	A	∞	-	-	-	-	-	-	-	< 0.95
9	7.68	10.13-5.23	AC AD AF	0.55	0.11	0.30	0.38	0.05	0.68	0.08	-	< 0.95
10	5.62	8.06-3.15	A	∞	0.09	-	-	-	-	-	-	0.99
11	4.12	6.32-2.04	A	∞	0.12	-	-	-	-	-	-	< 0.95
12	3.10	4.93-1.33	A	∞	0.18	-	-	-	-	-	-	0.99
13	2.20	3.79-0.85	A	∞	0.33	-	-	-	-	-	-	0.96
14	1.50	2.83-0.42	A	∞	1	-	-	-	-	-	-	< 0.95
15	4.17	6.43-2.05	A	∞	-	-	-	-	-	-	-	0.99
16	2.70	4.40-1.22	A	∞	-	-	-	-	-	-	-	1.00
17	1.99	3.36-0.82	A	∞	-	-	-	-	-	-	-	0.97
18	1.30	2.37-0.34	A	∞	-	-	-	-	-	-	-	0.99
19	1.13	2.17-0.22	A	∞	-	-	-	-	-	-	-	0.98
20	7.38	-	F CF DF	0.2	0.09	0.41	0.62	0.05	1.13	0.09	-	< 0.95
21	5.88	8.46-3.54	D CD DF	0.07	0.05	0.6	1.7	-	0.18	-	-	< 0.95
22	5.28	-	C D CD CF	0.09	0.07	1.31	0.71	-	0.33	-	-	< 0.95
23	2.52	5.34-0.32	CF DF	-	-	1	1	-	1	-	-	< 0.95
24	4.51	6.53-2.54	C CD	0.12	0.09	1.79	0.48	-	-	-	-	1.00
25	4.04	5.89-2.25	D CD	0.18	0.12	0.58	1.6	-	-	-	-	< 0.95
26	3.28	4.86-1.76	C AD CD	0.33	0.18	1.6	0.33	-	-	-	-	1.00
27	2.50	3.94-1.13	C AC	1	0.33	3	-	-	-	-	-	0.99
28	2.06	-	A AC BC	∞	1	1	-	-	-	-	-	< 0.95
29	2.27	3.57-1.09	CD	-	-	1	1	-	-	-	-	1.00
30	1.63	2.73-0.67	C	-	-	∞	-	-	-	-	-	0.99
31	0.77	1.49-0.15	C	-	-	∞	-	-	-	-	-	1.00
32	6.78	9.24-4.47	F	0.09	0.09	0.09	0.12	0.07	2.84	0.11	-	< 0.95
33	1.85	4.35-0.23	F	-	-	-	-	-	∞	-	-	0.96
34	6.54	8.96-4.29	F	0.12	0.12	0.12	0.18	0.09	1.88	0.15	-	< 0.95
35	5.81	-	F	0.18	0.18	0.18	-	-	4.56	0.07	-	< 0.95
36	3.88	6.34-1.63	F	-	-	-	-	-	∞	-	-	< 0.95
37	5.37	-	F	0.33	0.33	0.33	-	-	2.54	0.09	-	< 0.95
38	4.05	-	F	1	1	1	-	-	1	-	-	< 0.95
39	4.93	-	F	-	-	-	-	-	8.32	0.12	-	< 0.95
40	3.97	6.08-1.97	F	-	-	-	-	-	5.55	0.18	-	< 0.95
41	1.92	3.59-0.60	F	-	-	-	-	-	∞	-	-	1.00
42	3.31	-	F	-	-	-	-	-	3	0.33	-	< 0.95
43	2.61	-	FG	-	-	-	-	-	3	0.33	-	< 0.95
44	1.22	2.61-0.15	G	-	-	-	-	-	-	∞	-	1.00
45	4.53	6.42-2.78	F	-	-	-	0.33	0.12	1.16	0.18	-	1.00
46	3.57	5.18-2.00	F	-	-	-	-	-	∞	-	-	< 0.95
47	2.90	4.50-1.24	F	-	-	-	-	-	∞	-	-	< 0.95
48	2.03	3.57-0.61	F	-	-	-	-	-	∞	-	-	0.98
49	2.00	3.26-0.86	F	-	-	-	-	-	∞	-	-	1.00
50	0.83	1.64-0.16	F	-	-	-	-	-	∞	-	-	1.00
51	4.09	-	DF	-	-	-	1.00	0.18	0.18	0.33	-	< 0.95
52	3.88	-	FG	-	-	-	-	0.33	0.33	1	-	< 0.95
53	1.13	2.31-0.22	G	-	-	-	-	-	-	∞	-	1.00
54	0.55	1.27-0.04	G	-	-	-	-	-	-	∞	-	0.99
55	3.67	-	EF	-	-	-	-	1	1	-	-	< 0.95
56	2.82	4.59-1.26	F	-	-	-	-	-	∞	-	-	0.99

Node numbers refer to Figure 3. Posterior mean ages and 95% HPD intervals of divergence times are in millions of years before the present. DIVA reconstructions are shown for maxareas = 2. WAAA reconstructions are shown with probability indices (PI). Biogeographical areas are indicated by capital letters (as defined in Figure 1). Bayesian PP are also indicated.

### Historical biogeography of *Pseuduvaria*

**Figure 4:**
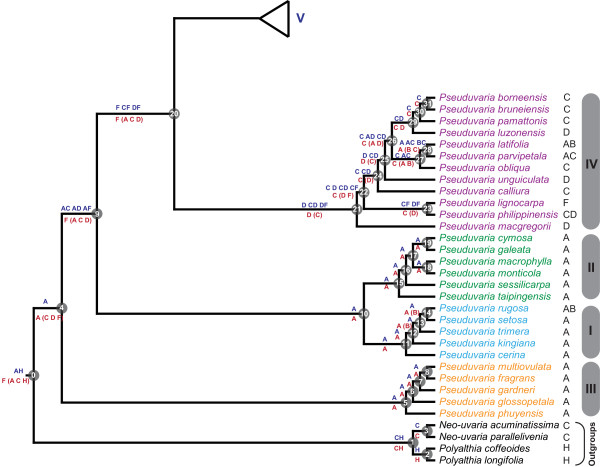
**Mapping of ancestral distributions on *Pseuduvaria***. Blue letters (above lines) represent DIVA results, and red letters (below lines) are WAAA results. For the latter results, red letters in brackets represent areas with lower probability indices. Letters to the right of the species names represent current species distributions (as defined in Figure 1).

**Figure 5:**
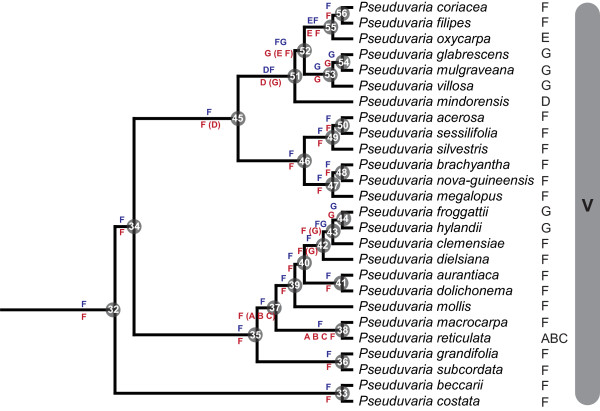
**Mapping of ancestral distributions on *Pseuduvaria *(continued)**. Blue letters (above lines) represent DIVA results, and red letters (below lines) are WAAA results. For the latter results, red letters in brackets represent areas with lower probability indices. Letters to the right of the species names represent current species distributions (as defined in Figure 1).

The BEAST tree generated using matrix B was used as input for DIVA and WAAA (results summarized in Table 3). The DIVA and WAAA results were largely congruent: the inferred ancestral distributions are shown on the tree (Figs 4, 5). The DIVA reconstructions required at least 20 dispersal events to explain the present-day distribution when the maximum number of areas was restricted to two at each node.

Both DIVA and WAAA indicate that the most likely ancestral area for *Pseuduvaria *was Sundaland (Figure 4: node 4). Diversification of *Pseuduvaria *within Sundaland gave rise to clades I, II and III. The inferred ancestral area of the combined clade IV-V was ambiguous, however (Figure 4: node 20): DIVA indicated that the ancestral area was either New Guinea, Borneo-New Guinea, or Philippines-New Guinea; whereas WAAA suggested that it was either New Guinea (with the highest PI of 1.13; Table 3) or the Philippines (PI = 0.62). It is noteworthy, however, that node 20 lacks statistical support.

The inferred ancestral area for the common ancestor of clade IV (Figure 4: node 21) is also ambiguous. DIVA indicated the inferred distribution was either the Philippines, Borneo-Philippines, or Philippines-New Guinea; whereas WAAA indicated that the Philippines was the most probable ancestral area, with the highest PI of 1.70 (Table 3). Within clade IV, a series of dispersal events could have occurred between Borneo and the Philippines, and also possibly between the Philippines and New Guinea. Furthermore, reverse westwards movements of *Pseuduvaria *from Borneo and/or the Philippines can also be inferred (Figure 4: node 28), as shown by the distribution of two species, *P. latifolia *and *P. parvipetala*, in Java and Sumatra.

The DIVA and WAAA results were congruent regarding the inferred ancestral distribution of clade V, both indicating that New Guinea was the most likely region (Figure 5: node 32). Subsequent speciation events occurred within New Guinea, which became the secondary centre of diversity in *Pseuduvaria*. Within clade V, dispersal events presumably occurred from New Guinea to Australia, although a westward migration also seems to have occurred from New Guinea to western Malesia, as evident in one species (*P. reticulata*).

## Discussion

### Historical biogeography of major Annonaceae clades

The divergence times within the Annonaceae differ slightly from previous age estimates based on NPRS and PL methods as implemented in r8s (see Table 2). Our results are comparable, however, with previous BEAST analyses: the *Isolona*-*Monodora *clade is dated at 13.9 Mya (95% HPD: 24.3-4.4 Mya; Figure 2A: node 13) in this study, consistent with estimates of ca. 14 Mya in a previous study using BEAST [11].

Previous studies have suggested that the Annonaceae are likely to have originated in western Gondwana [7,9]. The break-up of the Gondwanan supercontinent into western Gondwana (Africa and South America) and eastern Gondwana (Australia, Antarctica, Madagascar and India) began 180-150 Mya [48,49], but it was not until 90-85 Mya that the two became fully separated [50]. Biotic interchange between South America and Africa had essentially ceased by the latter half of the Late Cretaceous (80-65 Mya) [48,49], although it has been suggested that limited connection between the two continents was possible for sometime afterwards via the island chains of the Rio Grande Rise-Walvis Ridge and Sierra Leone Rises [51]. The earliest divergence in the Annonaceae appears to have occurred between 98.0 Mya (95% HPD: 101.5-94.9 Mya; Figure 2A: node 1) and 89.5 Mya (Figure 2A: calibrated node C2 at a minimum age of 89 Mya for the oldest known crown group fossil, *Futabanthus*), probably after the separation of the two landmasses.

*Anaxagorea *is shown to be sister to all other members of the Annonaceae. The age of the split between the *Cananga-Cyathocalyx-Cleistopholis*-*Tetrameranthus *clade and the combined LBC-SBC clade is estimated at 74.7 Mya (95% HPD: 84.4-63.6 Mya, PP = 1.00; Figure 2A: node 2). In previous studies this node has been used as a minimum age constraint of 68 Mya. The split age estimated here may correspond to the origin of seeds of the *Anonaspermum *type, and our results furthermore indicate that the age of this node could be older than 68 Mya. The tMRCA of the *Cananga-Cyathocalyx-Cleistopholis*-*Tetrameranthus *clade is estimated at 41.8 Mya (95% HPD: 62.4-22.3 Mya, PP = 1.00; Figure 2A: node 4). The genera in this clade are currently distributed in geographically disparate regions: *Cananga *occurs in Southeast Asia and Australia [52]; *Cyathocalyx *in Southeast Asia, extending east to Fiji [53,54]; *Cleistopholis *in Africa [52]; and *Tetrameranthus *in South America [55]. The ancestors of this clade may have dispersed between Africa, Asia and South America from 74.7 Mya (95% HPD: 84.4-63.6 Mya, PP = 1.00; Figure 2A: node 2) to 41.8 Mya (95% HPD: 62.4-22.3 Mya, PP = 1.00; Figure 2A: node 4). This disjunct distribution may have been the result of Eocene and early Oligocene cooling [7,56].

The LBC and SBC constitute over 90% of all Annonaceae genera. The age of the split between the LBC and SBC lineages is estimated at 67.3 Mya (95% HPD: 78.1-55.2 Mya, PP = 1.00; Figure 2A: node 3), and the mean tMRCA of the LBC at 59.6 Mya (95% HPD: 70.5-48.1 Mya, PP = 1.00; Figure 2A: node 6). The ancestors of the majority of LBC genera (*Guatteria*-*Asimina*) appear to have evolved ca. 51.5 Mya (95% HPD: 62.8-40.3 Mya, PP = 1.00; Figure 2A: node 8). These genera are mainly distributed in South America and Africa, although several occur in Asia, including *Artabotrys*, *Goniothalamus*, *Uvaria *and *Xylopia*; this suggests that the LBC originated in South America and/or Africa-Madagascar [[5]: Figure 5]. The wide geographical distribution of boreotropical taxa could be associated with the combined effects of plate tectonics and global climatic changes. The warming period during the late Paleocene-early Eocene thermal maximum, which peaked around the early Eocene Climatic Optimum (52-50 Mya) [57], may have promoted the northward dispersal of tropical plants [51]. This coincides with the suggested date for the clade consisting of all members (node 8) of the LBC except the basal *Mkilua*-*Trigynaea *lineage in the early Eocene. In a recent paleontological review, most modern subgroups of mammalian orders were shown to have appeared during the Paleocene-Eocene thermal maximum, suggesting that rapid environmental changes may have evolutionary significance [58]. During this period (ca. 56 Mya: [59]), a Greenland land bridge may have connected North America and Eurasia, enabling plant dispersals between the two continents [51]. Another possible dispersal route was across the Tethys seaway between Africa-Arabia and Asia, allowing tropical plant dispersal until the late Eocene-early Oligocene [56].

The mean tMRCA of the SBC is estimated at 39.8 Mya (95% HPD: 55.2-26.9 Mya, PP = 1.00; Figure 2A: node 7). The ancestral biogeographical origin of the SBC cannot be postulated here without a more extensive sampling of genera, although the inferred areas could be Africa-Madagascar as shown in the *Annickia*-*Piptostigma*-*Greenwayodendron *clade [[5]: Figure 5]. Most evolutionary diversifications within the SBC seemed to have initiated ca. 29.1 Mya (95% HPD: 39.2-20.2 Mya, PP = 1.00; Figure 2A: node 15). The rate of molecular evolution during the early divergence of the SBC (arrowed in Figure 2B, indicated by blue line) is about four times slower than that during the early divergence of the LBC (arrowed in Figure 2B, shown as thick red line).

The SBC, as shown in Figure 2A, consists of small clades from Africa (a clade consisting *Annickia*, *Piptostigma*, and *Greenwayodendron*) and South to Central America (*Onychopetalum-Cremastosperma*), with all other members from Asia. Diversification within the SBC ca. 29.1 Mya (Figure 2A: node 15) resulted in a South American clade consisting of *Cremastosperma*, *Klarobelia*, *Malmea*, *Mosannona*, *Onychopetalum*, *Oxandra*, *Pseudomalmea *and *Pseudoxandra*. The descendents of SBC genera could have dispersed from South America into Asia (since *Monocarpia euneura *occurs in Borneo [60]) via Africa and the Indian Plate; this presumably occurred between 29.1 (95% HPD: 39.2-20.2 Mya, PP = 1.00; Figure 2A: node 15) and 23.0 Mya (95% HPD: 31.1-16.0 Mya, PP = 1.00; Figure 2A: node 18). The alternative dispersal scenario (from Africa into Asia via Europe) is less likely because most Asian Annonaceae occur in warm and moist tropical rainforests. It is therefore more probable that the SBC genera dispersed from Africa to Asia via India, and that the dispersal between Africa and India occurred before the collision between Asia and India. Many past biogeographical studies have postulated that the collision between Asia and India occurred 50–55 Mya [61-63]. More recent work [64], however, suggests that this Early Eocene event marks the collision of India's northern margin with a sub-equatorially located island arc (remnants of which are today preserved as the Dazhuqu arc terrain in southern Tibet), with the main continent-continent collision taking place ca. 34 Mya. However, the passage of the subcontinent may have taken the north-eastern corner of the block [65] close to western South-east Asia, and thus faunal and floral exchanges between the two geographical regions may have been possible from the late Paleocene (ca. 57 Mya) onwards [50].

### Phylogeny and historical biogeography of *Pseuduvaria*

The BEAST topology of *Pseuduvaria *(Figure 3) is similar to those resulting from previous ML and Bayesian analyses (see Figure 3 in [25]). Although the phylogenetic positions of *Pseuduvaria *species in clades I, II and III remain identical, there are nevertheless some minor topological discrepancies between the trees. In the present study, clade III is shown to be sister to all other representatives of *Pseuduvaria*, whereas in previous analyses [25] this position was occupied by clade I. This is unlikely to have a significant impact on biogeographical interpretations, however, because the species in clades I, II and III are all distributed in geographical regions that were formerly part of the Sunda landmass. Another difference between the present and previous analyses relates to the phylogenetic position of two New Guinea species, *P. becarrii *and *P. costata*. These species are shown to be sister to all other members of clade V in the present study, but were basal within clade IV in the previous analyses [25]; the position of these two species is not statistically supported, although we suggest that they are more likely to belong to clade V as this clade is predominantly composed of New Guinea species. Several species in clade V (*P. oxycarpa*, *P. filipes*, *P. coriacea*, *P. mindorensis*, *P. reticulata*, and *P. mollis*) are furthermore located in different positions in different analyses, but their positions lack statistical support. Again, this may have little effect on biogeographical interpretations as most species in the clade are from New Guinea (except *P. mindorensis *from the Philippines, *P. oxycarpa *from Sulawesi, and *P. reticulata*, which is widespread in Malesia).

#### Late Miocene origin in Sundaland

Biogeographical reconstructions using DIVA and WAAA both suggest that *Pseuduvaria *originated in Sundaland (Figure 4: node 4). Initial BEAST analyses based on matrix A (with a broad taxonomic sampling) gave an estimated tMRCA for the *Pseuduvaria *lineage of 8.0 Mya (95% HPD: 11.5-5.0 Mya, PP = 1.00; Figure 2A: node 20); this node was subsequently selected as one of the two secondary molecular calibration points for *Pseuduvaria *in analyses based on matrix B (Figure 3: C4). These later analyses suggest a late Miocene origin, with a mean tMRCA of 8.3 Mya (95% HPD: 11.0-5.8 Mya, PP = 1.00; Figure 3: node 4). The former estimation from matrix A analyses, with a mean age of 8.0 Mya, will be used in all subsequent discussions presented here since this age was assigned as a calibration prior to the tMRCA of *Pseuduvaria *for matrix B analyses.

Early divergence of *Pseuduvaria *could have occurred between 14.9 Mya (95% HPD: 20.2-9.7 Mya, PP < 0.95; Figure 2A: node 19) and 8.0 Mya (95% HPD: 11.5-5.0 Mya; Figure 2A: node 20) during the Miocene, possibly associated with the 'mid-Miocene climatic optimum'. This warming phase occurred ca. 17-15 Mya [[57]: Figure 2], and led to the extensive growth of megathermal vegetation throughout most regions in Sundaland [66]. Subsequent global cooling during the middle Miocene climate transition (14.2-13.8 Mya) gradually resulted in the reduction of sea surface temperatures and the expansion of the Antarctic ice-sheets [67], which continued until ca. 6 Mya, in the early Pliocene [57]. These cold global temperatures were associated with a decline in CO_2 _levels, affecting the productivity of terrestrial vegetation [68], and resulted in the contraction of moist megathermal vegetation to the tropics and the concomitant expansion of grasslands [66]. This was followed by a warming phase that continued until ca. 3.2 Mya [57]. This climatic change was reflected by a steady increase in rainforest diversity in Southeast Asia until the mid Pliocene [66,69]. Subsequent diversifications of *Pseuduvaria *gave rise to clade III at ca. 6.6 Mya (95% HPD: 9.5-3.8 Mya; Figure 3: node 5) and clades I-II collectively at ca. 5.6 Mya (95% HPD: 8.1-3.2 Mya; Figure 3: node 10); this might be linked to these global climatic oscillations, with evolutionary divergence promoted by the significantly warmer and wetter climate.

#### Diversification in Sundaland

Biogeographical reconstructions of the ancestral distributions of clades I-III using DIVA and WAAA suggest that all three clades originated in Sundaland (Figure 4: nodes 5, 11, 15). Clade III consists of five species, *P. phuyensis*, *P. glossopetala*, *P. gardneri*, *P. multiovulata*, and *P. fragrans*; the tMCRA is estimated at ca. 6.6 Mya (node 5 in Table 3 and Figure 3). Clades I and II originated at a similar time, with tMRCA estimates of 4.1 and 4.2 Mya, respectively (nodes 11 and 15 in Table 3 and Figure 3).

Species in clades I and II are mostly endemic to Peninsular Malaysia or Thailand, with the exception of four comparatively widespread species, viz.: *P trimera *(southern China, Vietnam, Myanmar and Thailand), *P. setosa *(Thailand and Peninsular Malaysia), *P. rugosa *(Myanmar, Thailand, Peninsular Malaysia, Sumatra and Java), and *P. macrophylla *(Peninsular Malaysia and Sumatra) [25]. *Pseuduvaria rugosa *is estimated to have diverged around 1.5 Mya (95% HPD: 2.8-0.4 Mya, PP < 0.95; Table 3: node 14), at a similar time to *P. macrophylla *at ca. 1.3 Mya (95% HPD: 2.4-0.3 Mya, PP = 0.99; Table 3: node 18). In the middle and late Miocene, land connected Sumatra-Java to mainland Southeast Asia [70]. Further reduction of sea levels during the Pleistocene (to a maximum depth of 120 m below the present level) exposed additional land area that connected Peninsular Malaysia, Sumatra, Java, and Borneo [71]. Tropical forests also gradually expanded, as shown by the arrival of orangutans in Java [72]. At that time, *P. macrophylla *and *P. rugosa *were likely to have been able to disperse unimpeded between Sumatra, Java and Peninsular Malaysia; *P. trimera *would similarly have been able to advance northwards into southern China. Seed dispersal of *P. rugosa *is known to be facilitated by orangutans [27].

#### Dispersal out of Sundaland

**Figure 6:**
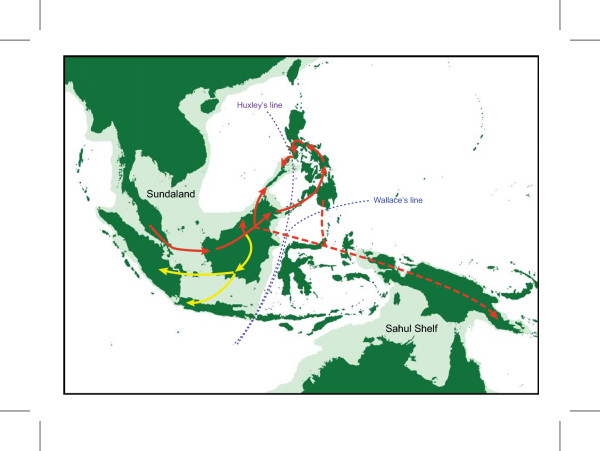
**Proposed biogeographical scenarios for *Pseuduvaria *clade IV**. Arrows represent possible migration routes (see text for details).

DIVA and WAAA indicated several possible ancestral distributions for clades IV and V collectively (Figure 4: node 20). According to DIVA, New Guinea, Borneo-New Guinea, or Philippines-New Guinea are potential ancestral areas, whereas WAAA highlighted New Guinea (PI = 1.13) and the Philippines (PI = 0.62). The age of node 9 is estimated at ca. 7.7 Mya, although this node is not statistically supported (PP < 0.95; Table 3). This age estimate (indicated by a dotted black line in Figure 3) could represent the earliest migration event for *Pseuduvaria *out of Sundaland to the Philippines and/or New Guinea (Figure 6).

The tMRCA of clade IV is estimated at ca. 5.9 Mya (95% HPD: 8.5-3.5 Mya, PP < 0.95; Figure 3: node 21). WAAA indicated that the Philippines is the most likely ancestral area, with the highest PI value of 1.7 (Table 3 and Figure 4: node 21), although Borneo also returned a relatively high PI of 0.6. Similar results were obtained using DIVA, with the Philippines or Borneo-Philippines as the most likely ancestral areas (the third suggested scenario, with New Guinea-Philippines as the ancestral area, is improbable since the two areas are not contiguous). The timing of dispersal events from Sundaland to the Philippines may be associated with the docking of the Philippine Sea Plate with the Sunda Block, which was initiated in the late Miocene-Pliocene [73].

The WAAA and DIVA results are complex within clade IV, but appear to show probable dispersals from Borneo and/or the Philippines eastwards into New Guinea (dotted red arrows in Figure 6) and westwards into continental Southeast Asia, Sumatra and/or Java (yellow arrows in Figure 6). The eastward migration into New Guinea may have occurred between node 22 (mean tMRCA: 5.3 Mya; without HPD, PP < 0.95; Table 3) and node 23 (mean tMRCA: 2.5 Mya; 95% HPD: 5.3-0.3 Mya, PP < 0.95; Table 3). These dispersals from Sundaland to New Guinea may have been achieved by three routes: island hopping after the collision of the Australian Plate with the Philippine Plate; stochastic dispersals across the Makassar Straits; or dispersal of montane taxa via island-hopping following the uplift of New Guinea and other islands [51]. The dispersal of *Dacrycarpus *(Podocarpaceae) is a suggested example of the third route: *Dacrycarpus *dispersed to New Guinea from Australia in the mid-Miocene and then dispersed to Borneo ca. 3.5 Mya in the mid-Pliocene [51]. *Pseuduvaria *species generally occur at low altitudes, suggesting that this third route is less likely to be important, although *P. lignocarpa *can occur at altitudes over 1000 m [27].

WAAA determined that the most probable ancestral areas at node 24 (Figure 4) are either Borneo (with the highest PI of 1.79; Table 3) or the Philippines (PI = 0.48; Table 3); similar results were obtained using DIVA. The ancestral distributions for node 25 (Figure 4) were the same as those for node 24, although node 25 is not statistically supported. A westward migration from Borneo-Philippines into mainland Southeast Asia, Sumatra and/or Java may have occurred by nodes 26, 27 and/or 28 (yellow arrows in Figure 6).

#### Colonization of New Guinea via long-distance dispersal

Clade V contains almost half of the genus, with most species currently distributed in New Guinea. The earliest suggested date for the colonization of New Guinea by *Pseuduvaria *species is 7.7 Mya (95% HPD: 10.1-5.2 Mya, PP < 0.95; Figure 4: node 9), although this clade lacks statistical support (Table 3). If colonization of New Guinea by node 9 is accepted, it is necessary to invoke a subsequent reverse westward dispersal for clades I and II (Figure 4: node 10). A more conservative scenario involves a later colonization of New Guinea after 7.4 Mya (Figure 4: node 20), although this node is also unsupported.

A recent plate tectonic modeling of New Guinea during the Cenozoic [[74]: Figure 8] has shown major changes in the motion of the Pacific Plate beginning around 43 Mya (Eocene), with the initiation of two subduction systems, generating the Inner and Outer Melanesian arcs. During the Oligocene (ca. 35-30 Mya), the first significant collision event began and the northern part of the Australia plate was subducted underneath the Inner Melanesian arc, creating the 'Peninsular orogeny' and generating the Papuan Peninsula [74]. As a result of a drop in sea level (ca. 90 m) during the Oligocene, central and western New Guinea became largely sub-aerial.

**Figure 7:**
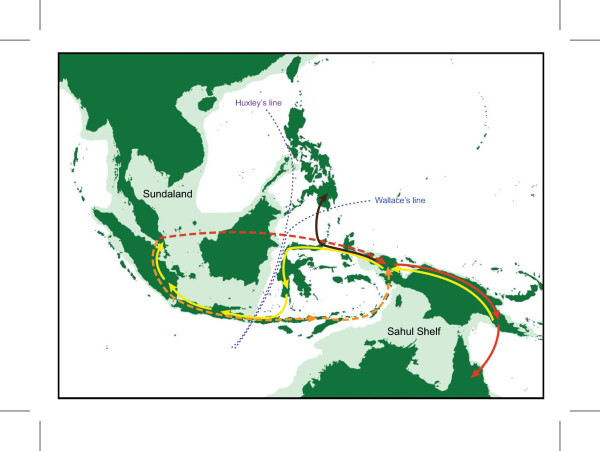
**Proposed biogeographical scenarios for *Pseuduvaria *clade V**. Arrows represent possible migration routes (see text for details).

In the late Miocene (ca. 10 Mya), a second collision event initiated in western New Guinea, creating the 'Central Range orogeny' [74]. At that time, the Sunda and the Sahul Shelves may have reached closest proximity, although floristic migration may have been unlikely since New Guinea was still largely submerged, and deep ocean barriers existed with no land bridges [75,76]. The best opportunity for biota to 'island hop' across Wallacea appears to have been during the last 5 million years [75], due to: (1) the formation of a substantial landmass in Sulawesi from ca. 5 Mya [75]; (2) the connection between New Guinea and Sundaland via the Banda and Sunda arcs [[70], p. 122]; (3) the increasing number of volcanic islands in East Indonesia [77]; (4) the exposure of the Sunda and Sahul Shelves caused by falling sea level (up to 120 m lower than present levels) during the Pleistocene glaciations [71]; and (5) the accretion of microcontinental island arc fragments at the northern edge of the Australian craton portion of New Guinea [75,77]. Migration of *Pseuduvaria *from Sundaland to New Guinea may therefore have been facilitated by a series of stepping stones, possibly via Borneo (dotted red arrow in Figure 7) or the Lesser Sunda Islands (dotted orange arrow in Figure 7). *Pseuduvaria *also occurs in the Aru islands (*P. aurantiaca*) and New Britain (*P. macrocarpa*). Similar biogeographical patterns are found in other plant groups such as the tribe Aglaieae (Meliaceae) which originated in western Malesia and subsequently dispersed eastwards to the Aru and Kai islands, New Guinea, New Britain, Australia, Fiji and other Pacific islands [78]; migration between the Sunda and Sahul shelves was likely to have been achieved via long-distance dispersal by birds [78].

#### Diversification patterns in New Guinea and Australia

*Pseuduvaria *may have begun to diversify in New Guinea after node 34 from ca. 6.5 Mya (95% HPD: 9.0-4.3 Mya, PP < 0.95; Figure 3), resulting in two main lineages with tMRCAs of ca. 5.8 Mya (Table 3: node 35) and ca. 4.5 Mya (Table 3: node 45). Coincident with this timeframe, there was a gradual transition between global cooling and warming phases: the cooling phase lasted until ca. 6 Mya, followed by a warming phase with moist climate until ca. 3.2 Mya [57]. A cooling period subsequently started again with a drier climate in the late Pliocene [79], followed by several cycles of glacial-interglacial episodes from ca. 1.8 Mya during the Pleistocene [80,81]. These climatic oscillations, combined with the changes in sea level, may have promoted *Pseuduvaria *speciation in New Guinea.

*Pseuduvaria *exhibits a high level of endemism (95%) in New Guinea [27]. This could be associated with the Central Range orogeny in New Guinea, which developed from ca. 8 Mya; this collisional orogenesis led to the formation of a ca. 1300 km-long mountainous backbone from the Bird's Neck to the Papuan Peninsula [74] with some peaks over 5000 m [82]. Speciation within New Guinea may therefore have been promoted by mountain ranges over 4000 m acting as physical barriers and influencing temperatures, and a continuously hot and wet climate with rainfalls of over 2500 mm per annum [83]. *Pseuduvaria *species in New Guinea diversified morphologically in response to these physical and climatic barriers, with evolutionary shifts from unisexual into bisexual flowers, elongation of flowering and fruiting peduncles (favouring fruit-bat frugivory), and the evolution of distinct pollination systems [25,27]. The New Guinea orogeny is analogous to the Miocene uplift of the Andean range, where different elevations (lowland or highland habitats) have impacted species diversification in the Neotropics [84].

There is also evidence of dispersal of *Pseuduvaria *species from New Guinea to northern Australia (Figs 5, 7), as shown in node 44 (*P. froggattii *and *P. hylandii*) and node 53 (*P. villosa*, *P. mulgraveana *and *P. glabrescens*). These clades are estimated to have evolved after 2.6 Mya (Table 3: node 43) and 3.9 Mya (Table 3: node 52), respectively. *Pseuduvaria *would have been able to disperse between New Guinea and Australia during the Pleistocene (red arrows in Figure 7): glaciations caused a lowering of oceans to at least 120 m below the present level [85-87], and as a result New Guinea and northern Australia formed a continuous land mass [76,86] with open forest vegetation growing across the Torres Strait land bridge (between eastern New Guinea and northern Australia) between 17,000 and 8,000 years ago [88].

Although clade V is largely composed of New Guinea and Australian species, there are three significant exceptions. *Pseuduvaria reticulata *is widespread in Peninsular Malaysia, Sumatra, Java, Lesser Sunda islands and Borneo, indicating an apparent westwards recolonization of Sundaland between 5.4 Mya (Table 3: node 37) and 4.1 Mya (Table 3: node 38). The most likely migration route from the Sahul Shelf to Sundaland (Java) may have been be achieved via Sulawesi [79] (yellow arrows in Figure 7); dispersal across the wide Makassar Straits seems unlikely since these straits have been shown to be an effective barrier between eastern and western Malesia [89].

A northward migration from New Guinea to the Philippines, possibly via Sulawesi (brown arrow in Figure 7), is shown by *P. mindorensis *after ca. 4.1 Mya (Table 3: node 51), although this node lacks statistical support. Another westwards reversal event is evident in *P. oxycarpa*, which may have migrated from New Guinea to Sulawesi between 3.9 (Table 3: node 52) and 3.7 Mya (Table 3: node 55). A substantial landmass was present in the Sulawesi region between ca. 10-5 Mya [75], but it was not until the last 5 Ma that the three distinct fragments (peninsular NE, mainland NE, and W Sulawesi) became united [90], facilitating the migration of plants and animals across Wallacea [75].

## Conclusion

The divergence times of the main clades within the Annonaceae (derived here using BEAST) deviate slightly from those previously determined using different calibration points and dating methods. In particular, our estimate for the SBC crown is 39.8 Mya (95% HPD: 55.2-26.9 Mya), which is much younger than previous estimates of 62.5-53.1 ± 3.6 Mya [7] and 58.76 Mya [8]. The present study differs from previous research as it utilizes an uncorrelated lognormal relaxed clock model and is calibrated using the recently described fossil, *Futabanthus*; these factors are likely to enable more precise divergence time estimates within the Annonaceae.

*Pseuduvaria *is shown to have originated in Sundaland in the late Miocene, ca. 8 Mya (95% HPD: 11.5-5.0 Mya). Subsequent migration events were predominantly eastwards towards New Guinea and Australia, although several migratory reversals are also postulated. The suggested dispersal episodes are broadly consistent with the available geological data.

The migration of *Pseuduvaria *species into New Guinea from Sundaland and/or the Philippines is of particular biogeographical interest. DIVA and WAAA reconstructions of the most probable ancestral areas at each node suggest that the earliest migration of *Pseuduvaria *species into New Guinea may have occurred as early as 7.7 Mya (95% HPD: 10.1-5.2 Mya), although this node is not statistically supported. If such a scenario is correct, it is necessary to invoke long-distance dispersal events since the geological data suggest that biotic migration between the two landmasses would have been unlikely: New Guinea was largely submerged during this period and the Sunda and Sahul landmasses were separated by deep ocean barriers without land bridges [75,76]. It was only within the past 5 Ma that significant opportunities may have existed for biotic migration into New Guinea from Sundaland [75], and significantly all the other postulated migrations between New Guinea and Sundaland occurred within this period: from Sundaland to New Guinea between nodes 22 and 23 in clade IV (i.e., after 5.3 Mya); from New Guinea to Sundaland between nodes 37 and 38 in clade V (i.e., after 5.4 Mya); and from New Guinea to Sulawesi between nodes 52 and 55 in clade V (i.e., after 3.9 Mya).

Our previous study of morphological character evolution within *Pseuduvaria *[25] indicated that the ancestors of clade V were likely to have had fruits with large monocarps (individual fruit segments derived from the maturation of individual carpels within the flower); this inference holds true irrespective of whether the New Guinea species *P. beccarii *and *P. costata *are included in clade IV (as indicated in our previous study [25]) or included in clade V (present analysis). There is a dearth of observational reports on frugivory and seed dispersal of *Pseuduvaria *species [27], although it appears that seeds of smaller-fruited species are bird-dispersed whereas those of larger-fruited species are dispersed by primates or fruit bats. It is probable that the long-distance dispersal events hypothesised to explain the early colonization of New Guinea by *Pseuduvaria *species may have been facilitated by fruit bats. Significantly, the majority of New Guinea species of *Pseuduvaria *possess elongate flowering peduncles and/or pedicels [25]: separation of fruits from the foliage (thereby ensuring that the fruits are conspicuous) is a common feature of plants adapted for fruit-bat frugivory [90].

## Authors' contributions

YCFS collected and analysed the molecular data. RMKS conceived the study and obtained funding. Both authors wrote the paper and have read and approved the final manuscript.

## Supplementary Material

Additional file 1 **Specimens and GenBank information**. Information regarding species sampling, localities, and GenBank accession numbers for sequences used in BEAST analyses. Click here for file
