# Supplementary material for: Evolutionary divergence times in the Annonaceae: evidence of a late Miocene origin of Pseuduvaria in Sundaland with subsequent diversification in New Guinea
Source: BMC Evol Biol. 2009 Jul 2;9:153. doi: 10.1186/1471-2148-9-153 (PMC2722625; doi:10.1186/1471-2148-9-153)
Supplement: Additional file 1 — Specimens and GenBank information. Information regarding species sampling, localities, and GenBank accession numbers for sequences used in BEAST analyses. [file 1471-2148-9-153-S1.doc]

Additional file 1. Information regarding species sampling, localities, and GenBank accession numbers for sequences used in BEAST analyses.

| Species | Collection information | Locality | *psbA-trnH* spacer | *trnL-F* | *matK* | *rbcL* | *atpB-rbcL* spacer |
| --- | --- | --- | --- | --- | --- | --- | --- |
| *Coelocaryon preussii* Warb. | Wieringa, J.J. 3640 (WAG) | Gabon | — | AY743456 | AY743475 | AY743437 | — |
| *Eupomatia bennettii* F.Muell. | Chatrou, L.W. s.n. (U) | UUBC | — | DQ861842 | Unpublished sequence from L.W.Chatrou | DQ861790 | — |
| *Liriodendron chinense* Sargent | Chatrou, L.W. et al. 279 (U) | China | — | AY841670 | — | AY841593 | — |
| *Magnolia kobus* DC. | Chatrou, L.W. et al. 278 (U) | Japan | — | AY743457 | AY743476 | AY743438 | — |
| *Persea americana* Mill. | Chatrou, L.W. 479 (U) | UUBC | Unpublished sequence from L.W.Chatrou | AY841669 | Unpublished sequence from L.W.Chatrou | AY841592 | Unpublished sequence from L.W.Chatrou |
| **Basal grade** |  |  |  |  |  |  |  |
| *Anaxagorea silvatica* R.E.Fries | Maas, P.J.M. et al. 8836 (U) | Brazil | AY841427 | AY743458 | AY743477 | AY743439 | AY578140 |

| *Cananga odorata* (Lam.) Hook.f. & Thomson | Chatrou, L.W. et al. 93 (U) | Costa Rica | AY841431 | AY841680 | AY841394 | AY841602 | AY841372 |
| --- | --- | --- | --- | --- | --- | --- | --- |
| *Cleistopholis glauca* Engl. & Diels | Wieringa, J.J. et al. 3278 (WAG) | Gabon | AY841432 | AY841681 | AY841395 | AY841603 | AY841373 |
| *Cyathocalyx martabanicus* Hook.f. & Thomson | Mols, J.B. 11 (L) | Kabun Raya Bogor | DQ125120 | AY841683 | DQ125054 | AY841605 | EF179253 |
| **Long Branch Clade** |  |  |  |  |  |  |  |
| *Annona glabra* L. | Chatrou, L.W. 467 (U) | USA | DQ125116 | AY841673 | DQ125050 | AY841596 | EF179246 |
| *Artabotrys hexapetalus* (L.f.) Bhandari | Chatrou, L.W. 470 (U) | Cult., Utrecht Univesrity Botanic Garden | AY841429 | EF179317 | AY238962 | AY238953 | EF179249 |
| *Asimina angustifolia* A.Gray | Weerasooriya, A. s.n. (MISS) | USA | DQ125119 | AY841677 | DQ125053 | DQ124939 | EF179251 |
| *Asimina triloba* (L.) Dunal | Chatrou, L.W. 276 (U) | North America | AY841430 | AY743460 | AY743479 | AY743441 | EF179252 |
| *Cymbopetalum brasiliense* (Vell.) Benth. ex Baill. | Chatrou, L.W. 471 (U) | Cultivated at the Utrecht Univesrity Botanic Garden | DQ125121 | AY841686 | DQ125055 | AY841608 | EF179254 |
| *Dasymaschalon macrocalyx* Finet & Gagnep. | Keßler, P.J.A. 3199 (L) | Thailand | EF179313 | AY841688 | EF179277 | AY841610 | EF179255 |

| *Duguetia hadrantha* (Diels) R.E.Fr. | Chatrou, L.W. 181 (U) | Peru | DQ125123 | AY740573 | AY740541 | AY738161 | EF179258 |
| --- | --- | --- | --- | --- | --- | --- | --- |
| *Duguetia staudtii* (Engl. & Diels) Chatrou | Andel, T.R. van 3290 (U) | Cameroon | DQ125124 | AY740590 | AY740558 | AY738178 | EF179259 |
| *Fusaea peruviana* R.E.Fr. | Chatrou, L.W. 179 (U) | Peru | AY841436 | AY743464 | AY743483 | AY743445 | EF179260 |
| *Goniothalamus griffithii* Hook.f. & Thomson | Keßler, P.J.A. 3188 (L) | Thailand | DQ125125 | AY743465 | AY743484 | AY743446 | EF179261 |
| *Goniothalamus tapis* Miq. | Keßler, P.J.A. 3193 (L) | Thailand | DQ125126 | AY841700 | DQ125058 | AY841622 | EF179262 |
| *Guatteria aeruginosa* Standl. | Chatrou, L.W. 66 (U) | Costa Rica | DQ125136 | AY741007 | AY740909 | AY740958 | EF179264 |
| *Guatteria* aff. *sellowiana* Schltdl. | Lobão, A.Q. 557 (U) | Brazil | — | AY741052 | AY740954 | AY741003 | — |
| *Isolona campanulata* Engl. & Diels | Chatrou, L.W. 472 (U) | Cult., Utrecht Univesrity Botanic Garden | DQ125127 | EF179318 | AY238963 | AY238954 | EF179266 |
| *Letestudoxa bella* Pellegr. | Wieringa, J.J. 2797 (WAG) | Gabon | DQ125128 | AY841707 | DQ125059 | AY841629 | EF179267 |
| *Mkilua fragrans* Verdc. | Chatrou, L.W. 474 (U) | Cult., Utrecht Univesrity Botanic Garden | DQ861696 | AY841712 | DQ125060 | AY841634 | EF179268 |

| *Monanthotaxis whytei* (Stapf) Verdc. | Chatrou, L.W. 475 (U) | Cult., Utrecht Univesrity Botanic Garden | EF179315 | AY841713 | EF179278 | AY841635 | EF179269 |
| --- | --- | --- | --- | --- | --- | --- | --- |
| *Monodora myristica* (Gaertn.) Dunal | Chatrou, L.W. 477 (U) | Cult., Utrecht Univesrity Botanic Garden | DQ125129 | AY743466 | AY743485 | AY743447 | EF179270 |
| *Neostenanthera myristicifolia* (Oliv.) Exell | Wieringa, J.J. 3566 (WAG) | Gabon | DQ125130 | AY743467 | AY743486 | AY743448 | EF179271 |
| *Pseudartabotrys letestui* Pellegr. | Wieringa, J.J. 3273 (WAG) | Gabon | DQ125131 | AY841728 | DQ125061 | AY841650 | EF179272 |
| *Rollinia herzogii* R.E.Fr. | Chatrou, L.W. 347 (U) | Bolivia | DQ125132 | AY841734 | DQ125062 | AY841656 | EF179273 |
| *Trigynaea lanceipetala* D.M.Johnson & N.A.Murray | Chatrou, L.W. 234 (U) | Peru | — | AY743468 | AY743487 | AY743449 | EF179274 |
| *Uvaria lucida* Benth. | Botanische Tuinen 84GR00334 (U) | West African | AY841440 | EF179319 | AY238966 | AY238957 | EF179275 |
| *Xylopia peruviana* Baill. | Chatrou, L.W. 483 (L) | Cult., Utrecht Univesrity Botanic Garden | DQ125134 | EF179320 | AY238967 | AY238958 | EF179276 |

| **Short Branch Clade** |  |  |  |  |  |  |  |
| --- | --- | --- | --- | --- | --- | --- | --- |
| *Alphonsea kinabaluensis* J.Sinclair | Ridsdale DV-S-3048 (L) | Malaysia | — | AY319080 | AY518811 | AY318968 | — |
| *Annickia chlorantha* (Oliv.) Setten & Maas | Sosef, M.S.M. 1877 (WAG) | Gabon | AY841442 | AY841671 | AY841393 | AY841594 | AY841370 |
| *Cremastosperma brevipes* (DC.) R.E.Fries | Scharf, U. 76 (U) | French Guiana | AY841447 | AY743573 | AY743550 | AY743527 | AY841374 |
| *Cremastosperma cauliflorum* R.E.Fries | Chatrou, L.W. et al. 224 (U) | Peru | AY841448 | AY743565 | AY743542 | AY743519 | AY841375 |
| *Greenwayodendron oliveri* (Engl.) Verdc. | Jongkind, C.C.H. et al. 1795 (WAG) | Ghana | AY841465 | AY743470 | AY743489 | AY743451 | AY841377 |
| *Haplostichanthus longirostris* (Scheffer) Heusden | Takeuchi W. & D. Ama 15656 (L) | New Guinea | — | AY319091 | AY518826 | AY318979 | — |
| *Klarobelia inundata* Chatrou | Chatrou, L.W. et al. 205 (U) | Peru | AY841469 | AY743471 | AY743490 | AY743452 | AY841378 |
| *Malmea dielsiana* R.E.Fries | Chatrou, L.W. et al. 122 (U) | Peru | AY841473 | AY231288 | AY238964 | AY238955 | AY841379 |
| *Miliusa campanulata* Pierre | Chalermglin, P. 440407-11 (TISTR) | Thailand | — | AY319096 | AY518842 | AY318984 | — |
| *Miliusa horsfieldii* (Benn.) Pierre | Mols J.B. 1 (L) | Indonesia | — | AY319098 | AY518849 | AY318986 | — |

| *Mitrephora keithii* Ridl. | Keßler, P.J.A. 3190 (L); Middleton, D.J. et al. 877 (L) | Thailand | EU522122 | AY319108 | AY518857 | AY318995 | EU522343 |
| --- | --- | --- | --- | --- | --- | --- | --- |
| *Monocarpia euneura* Miq. | Slik, F. 2931 (L) | Borneo | AY841477 | AY319111 | AY518865 | AY318998 | AY841381 |
| *Mosannona costaricensis* R.E.Fries | Chatrou, L.W. et al. 90 (U) | Costa Rica | AY841479 | AY743496 | AY743503 | AY743510 | AY841382 |
| *Neo-uvaria acuminatissima* (Miq.) Airy Shaw | Ridsdale DV-SR-4671 (L) | Malaysia | — | AY319112 | AY518793 | AY318999 | — |
| *Neo-uvaria paralellivenia* (Boerl.) H.Okada & K.Ueda | Keßler, P.J.A. sub IV-H-73 (L) | Indonesia | — | AY319113 | AY518794 | AY319000 | — |
| *Onychopetalum periquino* (Rusby) D.M.Johnson & N.A.Murray | Chatrou, L.W. et al. 425 (U) | Bolivia | AY871485 | AY319179 | AY518876 | AY319065 | AY841383 |
| *Orophea celebica* Miq. | Keßler, P.J.A. PK 2953 (L) | Indonesia | — | AY319117 | AY518814 | AY319004 | — |
| *Orophea enterocarpa* Maingay ex Hook.f. | Chalermglin, P. 440403 (TISTR) | Thailand | — | AY319119 | AY518815 | AY319006 | — |
| *Oxandra longipetala* R.E.Fries | Chatrou, L.W. et al. 114 (U) | Costa Rica | — | AY841719 | — | AY841641 | — |
| *Piptostigma mortehani* De Wild. | Wieringa, J.J. et al. 2779 (WAG) | Gabon | AY841498 | AY743473 | AY743492 | AY743454 | AY841384 |

| *Polyalthia coffeoides* (Thwaites ex Hook.f. & Thomson) Thwaites | Ratnayake, R.M.C.S. 1/03 (HKU) | Sri Lanka | EU522123 | EU522178 | EU522233 | EU522288 | EU522344 |
| --- | --- | --- | --- | --- | --- | --- | --- |
| *Polyalthia korinti* (Dunal) Thwaites | Ratnayake, R.M.C.S. 2/03 (HKU) | Sri Lanka | EU522124 | EU522179 | EU522234 | EU522289 | EU522345 |
| *Polyalthia longifolia* (Sonn.) Thwaites | Ratnayake, R.M.C.S. 3/04 (HKU) | Sri Lanka | EU522125 | EU522180 | EU522235 | EU522290 | EU522346 |
| *Polyalthia suberosa* (Roxb.) Thwaites | Chatrou, L.W. 480 (U); Botanical Gardens Kaiserslautern s.n. | India; Sri Lanka | AY841502 | AY319152 | AY518833 | AY319038 | AY841386 |
| *Pseudomalmea diclina* (R.E.Fries) Chatrou | Chatrou, L.W. et al. 211 (U) | Peru | AY841506 | AY319128 | AY841398 | AY319068 | AY841388 |
| *Pseudoxandra lucida* R.E.Fries | Chatrou, L.W. et al. 213 (U) | Peru | AY841510 | AY319190 | AY319190 | AY319076 | AY841389 |
| *Sapranthus viridiflorus* G.E.Schatz | Chatrou, L.W. et al. 55 (U) | Costa Rica | AY841515 | AY319165 | AY743493 | AY319051 | AY841391 |
| *Tetrameranthus duckei* R.E.Fries | Stevenson, D.W. et al. 1002 (U) | Brazil | — | AY841736 | — | AY841658 | — |

| ***Pseuduvaria*** |  |  |  |  |  |  |  |
| --- | --- | --- | --- | --- | --- | --- | --- |
| *Pseuduvaria acerosa* Y.C.F.Su & R.M.K.Saunders | Womersley, J.S. NGF 43899 (L) | New Guinea | EU522126 | EU522181 | EU522236 | EU522291 | EU522347 |
| *Pseuduvaria aurantiaca* (Zipp. ex Miq.) Merr. | Van Balgooy, M.M.J. 6871 (L) | Aru Islands | EU522127 | EU522182 | EU522237 | EU522292 | EU522348 |
| *Pseuduvaria beccarii* (Scheff.) J.Sinclair | Koster, C. BW 13802 (L) | New Guinea | EU522128 | EU522183 | EU522238 | EU522293 | EU522349 |
| *Pseuduvaria borneensis* Y.C.F.Su & R.M.K.Saunders | Ashton, P.S. S 18415 (L) | Borneo | EU522129 | EU522184 | EU522239 | EU522294 | EU522350 |
| *Pseuduvaria brachyantha* Y.C.F.Su & R.M.K.Saunders | Rau, K. 451 (L) | New Guinea | EU522130 | EU522185 | EU522240 | EU522295 | EU522351 |
| *Pseuduvaria bruneiensis* Y.C.F.Su & R.M.K.Saunders | Sands, M.J.S. et al. 5813 (L) | Borneo | EU522131 | EU522186 | EU522241 | EU522296 | EU522352 |
| *Pseuduvaria calliura* Airy-Shaw | Lee, B. S 40622 (L) | Borneo | EU522132 | EU522187 | EU522242 | EU522297 | EU522353 |

| *Pseuduvaria cerina* J.Sinclair | Sinclair, J. 7634 (E) | Peninsular Malaysia | EU522133 | EU522188 | EU522243 | EU522298 | EU522354 |
| --- | --- | --- | --- | --- | --- | --- | --- |
| *Pseuduvaria clemensiae* Y.C.F.Su & R.M.K.Saunders | Takeuchi, W. & J. Regalado 10238 (L) | New Guinea | EU522134 | EU522189 | EU522244 | EU522299 | EU522355 |
| *Pseuduvaria coriacea* Y.C.F.Su & R.M.K.Saunders | Takeuchi, W. & D. Ama 15623 (L) | New Guinea | EU522135 | EU522190 | EU522245 | EU522300 | EU522356 |
| *Pseuduvaria costata* (Scheff.) J.Sinclair | Vink, W. 17573 (LAE) | New Guinea | EU522136 | EU522191 | EU522246 | EU522301 | EU522357 |
| *Pseuduvaria cymosa* (J.Sinclair) Y.C.F.Su & R.M.K.Saunders | Teo, L.E. & G. Pachiappan 857 (L) | Peninsular Malaysia | EU522137 | EU522192 | EU522247 | EU522302 | EU522358 |
| *Pseuduvaria dielsiana* (Lauterb.) J.Sinclair | Pullen, R. 1497 (L) | New Guinea | EU522138 | EU522193 | EU522248 | EU522303 | EU522359 |
| *Pseuduvaria dolichonema* (Diels) J.Sinclair | Takeuchi, W. & A. Towati 15517 (L) | New Guinea | EU522139 | EU522194 | EU522249 | EU522304 | EU522360 |
| *Pseuduvaria filipes* (Lauterb. & K.Schum.) J.Sinclair | Takeuchi, W. et al. 14061 (L) | New Guinea | EU522140 | EU522195 | EU522250 | EU522305 | EU522361 |

| *Pseuduvaria froggattii* (F.Muell.) Jessup | Ford, A. & L. Cinelli 4776 (HKU) | Australia | EU522141 | EU522196 | EU522251 | EU522306 | EU522362 |
| --- | --- | --- | --- | --- | --- | --- | --- |
| *Pseuduvaria fragrans* Y.C.F.Su, Chaowasku & R.M.K.Saunders | Chaowasku, T. 27 (L) | Thailand | EU522176 | EU522231 | EU522286 | EU522341 | EU522397 |
| *Pseuduvaria galeata* J.Sinclair | Sinclair, J. 10689 (L) | Peninsular Malaysia | EU522142 | EU522197 | EU522252 | EU522307 | EU522363 |
| *Pseuduvaria gardneri* Y.C.F.Su, Chaowasku & R.M.K.Saunders | Gardner S. & P. Sidisunthorn ST2313 (L) | Thailand | GQ174294 | GQ174296 | GQ174298 | GQ174300 | GQ174302 |
| *Pseuduvaria glabrescens* (Jessup) Y.C.F.Su & R.M.K.Saunders | Ford, A. 4672 (HKU) | Australia | EU522143 | EU522198 | EU522253 | EU522308 | EU522364 |
| *Pseuduvaria glossopetala* Y.C.F.Su & R.M.K.Saunders | Chin, S.C. 875 (L) | Peninsular Malaysia | GQ174295 | GQ174297 | GQ174299 | GQ174301 | GQ174303 |
| *Pseuduvaria grandifolia* (Warb.) J.Sinclair | Takeuchi, W. & D. Ama 15664 (L) | New Guinea | EU522144 | EU522199 | EU522254 | EU522309 | EU522365 |

| *Pseuduvaria hylandii* Jessup | Ford, A. & M. Bradford 4657 (HKU) | Australia | EU522145 | EU522200 | EU522255 | EU522310 | EU522366 |
| --- | --- | --- | --- | --- | --- | --- | --- |
| *Pseuduvaria kingiana* Y.C.F.Su & R.M.K.Saunders | Chung, R.C.K. et al. FRI 39471 (L) | Peninsular Malaysia | EU522146 | EU522201 | EU522256 | EU522311 | EU522367 |
| *Pseuduvaria latifolia* (Blume) Bakh.f. | Unknown collector (L [898.62-95]) | Java | EU522147 | EU522202 | EU522257 | EU522312 | EU522368 |
| *Pseuduvaria lignocarpa* J.Sinclair | Carr, C.E. 14304 (L) | New Guinea | EU522148 | EU522203 | EU522258 | EU522313 | EU522369 |
| *Pseuduvaria luzonensis* (Merr.) Y.C.F.Su & R.M.K.Saunders | de la Fuentes, R. 38732 (L) | Philippines | EU522149 | EU522204 | EU522259 | EU522314 | EU522370 |
| *Pseuduvaria macgregorii* Merr. | Soejarto, D.D. et al. 7985 (L) | Philippines | EU522150 | EU522205 | EU522260 | EU522315 | EU522371 |
| *Pseuduvaria macrocarpa* (Burck) Y.C.F.Su & R.M.K.Saunders | Takeuchi, W. et al. 13856 (L) | New Guinea | EU522151 | EU522206 | EU522261 | EU522316 | EU522372 |
| *Pseuduvaria macrophylla* (Oliv.) Merr. | de Wilde, W.J.J.O. & B.E.E. de Wilde-Duyfjes 19396 (L) | Sumatra | EU522152 | EU522207 | EU522262 | EU522317 | EU522373 |

| *Pseuduvaria megalopus* (K.Schum.) Y.C.F.Su & J.B.Mols | Takeuchi, W. & D. Ama 16235 (L) | New Guinea | EU522153 | EU522208 | EU522263 | EU522318 | EU522374 |
| --- | --- | --- | --- | --- | --- | --- | --- |
| *Pseuduvaria mindorensis* Y.C.F.Su & R.M.K.Saunders | Ridsdale, C.E. 883 (L) | Philippines | EU522154 | EU522209 | EU522264 | EU522319 | EU522375 |
| *Pseuduvaria mollis* (Warb.) J.Sinclair | Womersley, J.S. NGF 43718 (L) | New Guinea | EU522155 | EU522210 | EU522265 | EU522320 | EU522376 |
| *Pseuduvaria monticola* J.Sinclair | Chew, W.L. 849 (L) | Peninsular Malaysia | EU522156 | EU522211 | EU522266 | EU522321 | EU522377 |
| *Pseuduvaria mulgraveana* Jessup | Ford, A. 4658 (HKU) | Australia | EU522157 | EU522212 | EU522267 | EU522322 | EU522378 |
| *Pseuduvaria multiovulata* (C.E.C.Fisch.) J.Sinclair | Chalermglin, P. 431228 (HKU) | Thailand | EU522158 | EU522213 | EU522268 | EU522323 | EU522379 |
| *Pseuduvaria nova-guineensis* J.Sinclair | Croft, J.R. et al. LAE 68763 (L) | New Guinea | EU522159 | EU522214 | EU522269 | EU522324 | EU522380 |
| *Pseuduvaria obliqua* Y.C.F.Su & R.M.K.Saunders | Sidiyasa, K. 2222 (L) | Borneo | EU522160 | EU522215 | EU522270 | EU522325 | EU522381 |

| *Pseuduvaria oxycarpa* (Boerl. ex Koord.) Y.C.F.Su & R.M.K.Saunders | de Vogel, E.F. & J. Vermeulen 6639 (L) | Sulawesi | EU522161 | EU522216 | EU522271 | EU522326 | EU522382 |
| --- | --- | --- | --- | --- | --- | --- | --- |
| *Pseuduvaria pamattonis* (Miq.) Y.C.F.Su & R.M.K.Saunders | Podzorski, A.C. SMHI 520 (L) | Philippines | EU522162 | EU522217 | EU522272 | EU522327 | EU522383 |
| *Pseuduvaria parvipetala* Y.C.F.Su & R.M.K.Saunders | Veldkamp, J.F. 8241 (L) | Borneo | EU522163 | EU522218 | EU522273 | EU522328 | EU522384 |
| *Pseuduvaria philippinensis* Merr. | Ramos, M. BSN 24449 (SING) | Philippines | EU522164 | EU522219 | EU522274 | EU522329 | EU522385 |
| *Pseuduvaria phuyensis* R.M.K.Saunders, Y.C.F.Su & Chalermglin | Saunders, R.M.K. et al. 99/2 (HKU) | Thailand | EU522121 | EU522177 | EU522232 | EU522287 | EU522342 |
| *Pseuduvaria reticulata* (Blume) Miq. | de Wilde, W.J.J.O. & B.E.E. de Wilde-Duyfjes 14562 (L) | Sumatra | EU522165 | EU522220 | EU522275 | EU522330 | EU522386 |
| *Pseuduvaria rugosa* (Blume) Merr. | Phusomsaeng, S. 47 (L) | Thailand | EU522166 | EU522221 | EU522276 | EU522331 | EU522387 |

| *Pseuduvaria sessilicarpa* (J.Sinclair) Y.C.F.Su & R.M.K.Saunders | Burkill, H.M. 1853 (SING) | Peninsular Malaysia | EU522167 | EU522222 | EU522277 | EU522332 | EU522388 |
| --- | --- | --- | --- | --- | --- | --- | --- |
| *Pseuduvaria sessilifolia* J.Sinclair | Craven, L.A. & R. Schodde 1043 (L) | New Guinea | EU522168 | EU522223 | EU522278 | EU522333 | EU522389 |
| *Pseuduvaria setosa* (King) J.Sinclair | Maxwell, J.F. 86-208 (L) | Thailand | EU522169 | EU522224 | EU522279 | EU522334 | EU522390 |
| *Pseuduvaria silvestris* (Diels) J.Sinclair | Rullen, R. 8371 (L) | New Guinea | EU522170 | EU522225 | EU522280 | EU522335 | EU522391 |
| *Pseuduvaria subcordata* Y.C.F.Su & R.M.K.Saunders | Katik, P. LAE 70801 (L) | New Guinea | EU522171 | EU522226 | EU522281 | EU522336 | EU522392 |
| *Pseuduvaria taipingensis* J.Sinclair | Burkill, I.H. & M. Haniff 13023 (SING) | Peninsular Malaysia | EU522172 | EU522227 | EU522282 | EU522337 | EU522393 |
| *Pseuduvaria trimera* (Craib) Y.C.F.Su & R.M.K.Saunders | Saunders, R.M.K. et al. 99/4 (HKU) | Thailand | EU522173 | EU522228 | EU522283 | EU522338 | EU522394 |

| *Pseuduvaria unguiculata* (Elmer) Y.C.F.Su & R.M.K.Saunders | Escritor, L. BS 20834 (L) | Philippines | EU522174 | EU522229 | EU522284 | EU522339 | EU522395 |
| --- | --- | --- | --- | --- | --- | --- | --- |
| *Pseuduvaria villosa* Jessup | Wells, J. 8 (HKU) | Australia | EU522175 | EU522230 | EU522285 | EU522340 | EU522396 |
| *Pseuduvaria* sp. | Chaowasku, T. 27 (L) | Thailand | EU522176 | EU522231 | EU522286 | EU522341 | EU522397 |
